# Development and Prospective Internal-Validation Protocol of the SpinePROTECT Score: A Digital Biopsychosocial Risk Stratification Framework for Spine Surgery

**DOI:** 10.3390/medicina62102027

**Published:** 2026-10-20

**Authors:** Christian Riediger, Mark Ferl, Christoph H. Lohmann, Maria Schönrogge

**Affiliations:** Department of Orthopaedic Surgery, University Hospital Magdeburg, Otto von Guericke University Magdeburg, Leipziger Strasse 44, 39120 Magdeburg, Germany

**Keywords:** spine surgery, biopsychosocial model, risk stratification, clinical prediction model, patient-reported outcome measures, Type-D personality, digital health, study protocol

## Abstract

*Background and Objectives*: Postoperative recovery after spine surgery remains heterogeneous, and conventional perioperative risk assessment insufficiently represents psychosocial determinants. We developed the preliminary SpinePROTECT Score within a digital biopsychosocial framework intended to support structured preoperative risk stratification. *Materials and Methods*: Framework development integrated a structured narrative evidence review, evidence from three complementary institutional research programs, predefined candidate-predictor criteria, expert-informed preliminary weighting, and translation into a modular digital platform. The protocol defines a prospective model-development and internal-validation cohort of 300 adults undergoing elective primary or revision surgery for spinal disorders, with follow-up at 6 weeks, 3 months, and 12 months. The study was prospectively registered in the German Clinical Trials Register on 8 September 2026 (DRKS00041544) following ethics approval on 28 August 2026 (protocol code 151/26). At prospective registration, recruitment was scheduled to begin on 10 September 2026; the present protocol article reports no prospective outcome data. *Results*: The development process yielded three complementary domains: psychological vulnerability, clinical burden, and procedural invasiveness. The preliminary framework includes Type-D personality, depression, anxiety, pain catastrophizing, fear-avoidance beliefs, baseline disability, symptom duration, opioid use, revision status, and surgical trauma. Digital implementation enables electronic questionnaire acquisition, automated calculation of the preliminary SpinePROTECT Score, standardized reports, longitudinal follow-up, and structured research-data capture. Predictive performance is not reported in this protocol article. *Conclusions*: The SpinePROTECT framework provides a transparent methodological and digital foundation for biopsychosocial risk stratification in spine surgery. The planned prospective study will estimate model coefficients and determine discrimination, calibration, internal validity, and clinical utility before the SpinePROTECT Score is considered for outcome prediction or treatment guidance.

## 1. Introduction

Postoperative outcome after spine surgery is influenced by both clinical and psychosocial factors, and multidimensional predictors may explain part of the marked variability in recovery [1,2]. Chronic pain and functional recovery are therefore appropriately interpreted within a biopsychosocial framework rather than through anatomical findings alone [3]. Contemporary perioperative spine care has also increasingly adopted enhanced-recovery pathways; a recent systematic review and meta-analysis found that enhanced recovery after surgery protocols were associated with shorter hospitalization, fewer perioperative complications, lower opioid consumption, and earlier mobilization [4]. Despite these advances, a clinically relevant proportion of patients continue to experience persistent pain, functional impairment, prolonged opioid use, or dissatisfaction after technically successful procedures [1,2].

Spinal disorders also represent a major population-level health burden. In the Global Burden of Disease 2021 analysis, low back pain affected an estimated 619 million people worldwide in 2020 and remained the leading cause of years lived with disability, with prevalence projected to increase further by 2050 [5]. Persistent pain after spinal surgery remains clinically relevant despite advances in operative care; a 2023 systematic review and meta-analysis of 16 studies including 85,643 patients estimated a pooled prevalence of persistent pain of approximately 15% [6]. Persistent or recurrent pain after spinal surgery has historically been described as failed back surgery syndrome (FBSS); contemporary terminology increasingly uses persistent spinal pain syndrome type 2 (PSPS-T2) to describe chronic axial and/or extremity pain persisting after spinal surgery [7]. These data emphasize the need to identify modifiable and non-modifiable determinants of unfavorable recovery before surgery.

Traditional preoperative assessment in spine surgery emphasizes clinical symptoms, neurological findings, radiological pathology, comorbidity burden, and anticipated procedural complexity [2]. These biomedical variables remain fundamental for surgical indication and perioperative risk assessment. However, psychosocial characteristics also contribute to variation in pain and functional outcomes after spine surgery [1,2]. This interaction between biological, psychological, behavioral, and treatment-related factors is consistent with the biopsychosocial model of chronic pain and rehabilitation [3,8].

Psychological vulnerability has repeatedly been associated with less favorable recovery after spine surgery [1,9,10,11]. Depression has been associated with poorer postoperative outcomes and higher rates of several medical and surgical complications [12]. Pain catastrophizing and fear-related cognitions are established pain-related psychological constructs that can contribute to disability and adverse recovery trajectories [13,14]. In cervical spine surgery, preoperative depression and anxiety have also been associated with patient-reported outcomes, healthcare payments, and opioid use [15]. Preoperative opioid exposure itself is associated with prolonged postoperative opioid use and worse outcomes after spine surgery [16].

Particular attention has recently been directed toward stable personality characteristics as determinants of postoperative recovery. Type-D (“distressed”) personality, originally described by Denollet, combines the personality traits of negative affectivity and social inhibition into a stable psychosocial phenotype associated with chronic emotional distress, maladaptive coping strategies, and impaired stress regulation [17]. Although Type-D personality has been extensively investigated in cardiovascular medicine, evidence regarding its role in spine surgery has only recently emerged. In a prospective cohort study conducted by our group, patients with Type-D personality demonstrated significantly poorer long-term improvement in pain, disability, and patient satisfaction after spine surgery compared with non-Type-D patients [18]. These observations were subsequently confirmed in a university spine outpatient cohort, in which approximately one-third of patients fulfilled the diagnostic criteria for Type-D personality and exhibited substantially higher levels of pain, disability, depression, anxiety, and psychosocial burden [19]. Together, these findings suggest that Type-D personality represents a clinically relevant marker of global psychosocial vulnerability rather than an isolated psychological characteristic.

Several prediction models have been developed to support perioperative risk assessment in spine surgery [20,21,22]. Depending on the model, predicted outcomes include complications, adverse events, frailty-associated risk, and patient-reported outcomes [20,21,22,23,24]. Reviews of this literature describe substantial heterogeneity in model populations, predictors, outcomes, and validation strategies, with external validation remaining limited for many models [20,21,22]. Psychological constructs are also inconsistently represented in existing spine prediction models [20,21]. Validated instruments such as the DS14, PCS, and FABQ quantify specific psychosocial domains [17,25,26], but their incorporation into integrated perioperative prediction frameworks remains uncommon [20,21].

Digital health and machine-learning approaches are increasingly being investigated for outcome prediction in spine surgery [22,27,28]. Such approaches can support standardized data acquisition, longitudinal outcome assessment, and algorithmic risk estimation, although external validation and clinical implementation remain important limitations [21,22]. Within the literature reviewed for the present framework, we did not identify a dedicated spine surgery platform that simultaneously integrates the prespecified psychological measures, clinical characteristics, and a standardized measure of surgical invasiveness used in SpinePROTECT; this observation should be interpreted as a finding of the present structured narrative review rather than as evidence from a formal systematic review.

To address this unmet clinical need, we developed the SpinePROTECT Score, a digital biopsychosocial risk stratification framework intended to complement conventional preoperative assessment in spine surgery. The framework was constructed through a structured methodological process integrating structured evidence synthesis with findings from three complementary clinical research programs investigating psychological vulnerability, Type-D personality, and surgical invasiveness. Rather than replacing established surgical indications, the SpinePROTECT framework aims to identify patients at increased risk of unfavorable postoperative recovery, thereby facilitating individualized perioperative counseling, risk-adjusted clinical pathways, and standardized patient stratification for future clinical research.

The objective of the present study is to describe the scientific rationale, methodological development, preliminary score architecture, digital implementation, and prospective model-development and internal-validation strategy of the SpinePROTECT framework. The report does not present a validated clinical prediction model.

## 2. Materials and Methods

### 2.1. Study Design and Reporting Framework

The preliminary SpinePROTECT Score was developed within a methodological framework for biopsychosocial perioperative risk stratification in spine surgery. The overall development process followed a structured, evidence-based approach integrating a structured narrative evidence review, original clinical research, expert consensus, and digital implementation. Development and reporting were informed by current recommendations for clinical prediction-model research, including TRIPOD+AI [29]. Candidate variables were derived from complementary sources of evidence and translated into a standardized digital research platform.

Development was performed in six consecutive phases:

- Structured narrative evidence review;
- Identification of candidate variables;
- Construction of biopsychosocial domains;
- Preliminary score weighting;
- Digital implementation; and
- Prospective model development and internal validation.

This structured framework was designed to maximize transparency, reproducibility, and future adaptability while allowing prospective optimization of individual score components during prospective model development and internal validation.

The present report describes the methodological development and the protocol for prospective model development and internal validation. Reporting was informed by TRIPOD+AI [29], and risk-of-bias considerations were informed by PROBAST [30]. Because model coefficients and performance estimates have not yet been derived from the prospective cohort, the preliminary point system is explicitly regarded as a prespecified, hypothesis-generating framework rather than a validated clinical prediction model.

### 2.2. Literature Review and Evidence Base

A structured narrative evidence review was conducted using PubMed, Embase, and Web of Science to identify studies investigating psychological, clinical, and procedural predictors of postoperative outcome after spine surgery. Search terms included combinations of spine surgery, psychological factors, depression, anxiety, catastrophizing, fear avoidance, patient-reported outcomes, prediction model and risk stratification. English-language original studies, systematic reviews, and meta-analyses were considered. The review was intended to inform candidate-predictor selection and was not conducted or reported as a formal systematic review. Candidate predictors were evaluated according to clinical relevance, consistency of evidence, feasibility, reproducibility, and compatibility with digital implementation.

#### Scientific Rationale and Evidence Base

**Figure 1:**
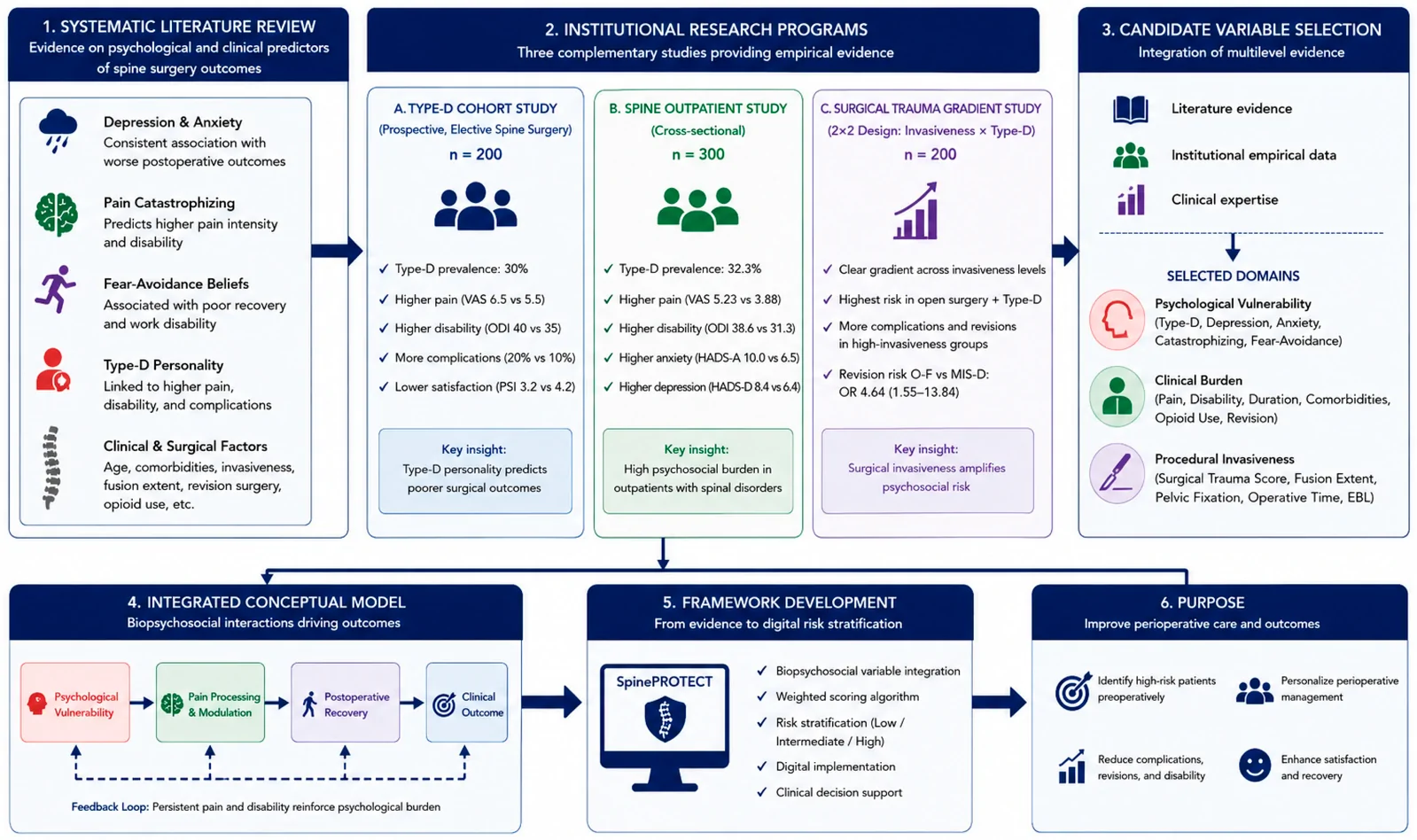
Scientific rationale underlying development of the SpinePROTECT framework. Development integrated structured evidence with institutional research on Type-D personality, psychosocial burden, and procedural invasiveness.

The scientific rationale underlying the SpinePROTECT framework was based on the integration of published evidence with three complementary institutional research programs investigating determinants of postoperative recovery in spine surgery (Figure 1).

First, published evidence has associated psychological distress with postoperative pain, disability, satisfaction, and other outcomes after spine surgery [1,9,10,11,12,15,31]. Pain catastrophizing and fear-avoidance constructs provide additional mechanistic and prognostic context for pain-related disability [25,26,32,33]. Together, these findings support the inclusion of psychosocial vulnerability within a biopsychosocial framework of postoperative recovery [3,8].

Second, a prospective observational cohort conducted at our institution investigated the influence of Type-D personality on postoperative recovery after spine surgery (n = 200). Patients fulfilling diagnostic criteria for Type-D personality demonstrated significantly less improvement in pain and disability together with higher complication rates and lower postoperative satisfaction than non-Type-D patients [18].

Third, a cross-sectional university spine outpatient cohort (n = 300) evaluated psychosocial burden in patients presenting with spinal disorders. Approximately one-third of patients fulfilled diagnostic criteria for Type-D personality and exhibited significantly higher pain intensity, disability, depression, anxiety, and overall psychosocial burden than non-Type-D patients [19].

Finally, procedural characteristics were incorporated through the previously developed Surgical Trauma Gradient, operationalized as the Surgical Trauma Score (STS), which quantified operative invasiveness as an independent determinant of postoperative recovery and complication risk [34].

Collectively, these complementary sources formed the scientific foundation of the SpinePROTECT framework.

### 2.3. Candidate-Predictor Selection

Selection of candidate variables integrated published evidence on psychosocial and clinical prognostic factors [1,9,10,11,12,13,14,15,16,25,26,31,32,33,35] with our previous clinical investigations on Type-D personality and procedural invasiveness [18,19,34]. Candidate variables were then assessed against predefined clinical and methodological criteria informed by the contemporary prediction-model literature [20].

Selection criteria included:

- Consistent association with postoperative outcome in the published literature;
- Availability of validated assessment instruments;
- Clinical relevance for perioperative decision-making;
- Feasibility within routine outpatient workflows;
- Compatibility with digital implementation;
- Potential for standardized longitudinal assessment.

Based on these criteria, three principal domains were established.

The psychological domain includes validated patient-reported outcome measures reflecting emotional distress, psychological vulnerability, pain-related cognition, and coping behavior. Instruments incorporated into the framework comprise the Type-D Scale (DS14) [17], Patient Health Questionnaire-9 (PHQ-9), Generalized Anxiety Disorder-7 (GAD-7), Pain Catastrophizing Scale (PCS) [25], and Fear-Avoidance Beliefs Questionnaire (FABQ) [26].

The clinical domain includes established patient-related predictors of postoperative recovery, including symptom duration, previous spine surgery, opioid consumption [16,22], and baseline disability measured using the Oswestry Disability Index (ODI) [33].

The surgical domain incorporates procedure-related variables representing biological surgical burden. Rather than considering isolated procedural characteristics independently, surgical complexity is summarized using the previously developed Surgical Trauma Gradient, operationalized as the Surgical Trauma Score (STS), allowing standardized classification of operative invasiveness [34].

The complete variable-selection framework is presented in Table 1.

### 2.4. Framework Construction and Preliminary Weighting

**Figure 2:**
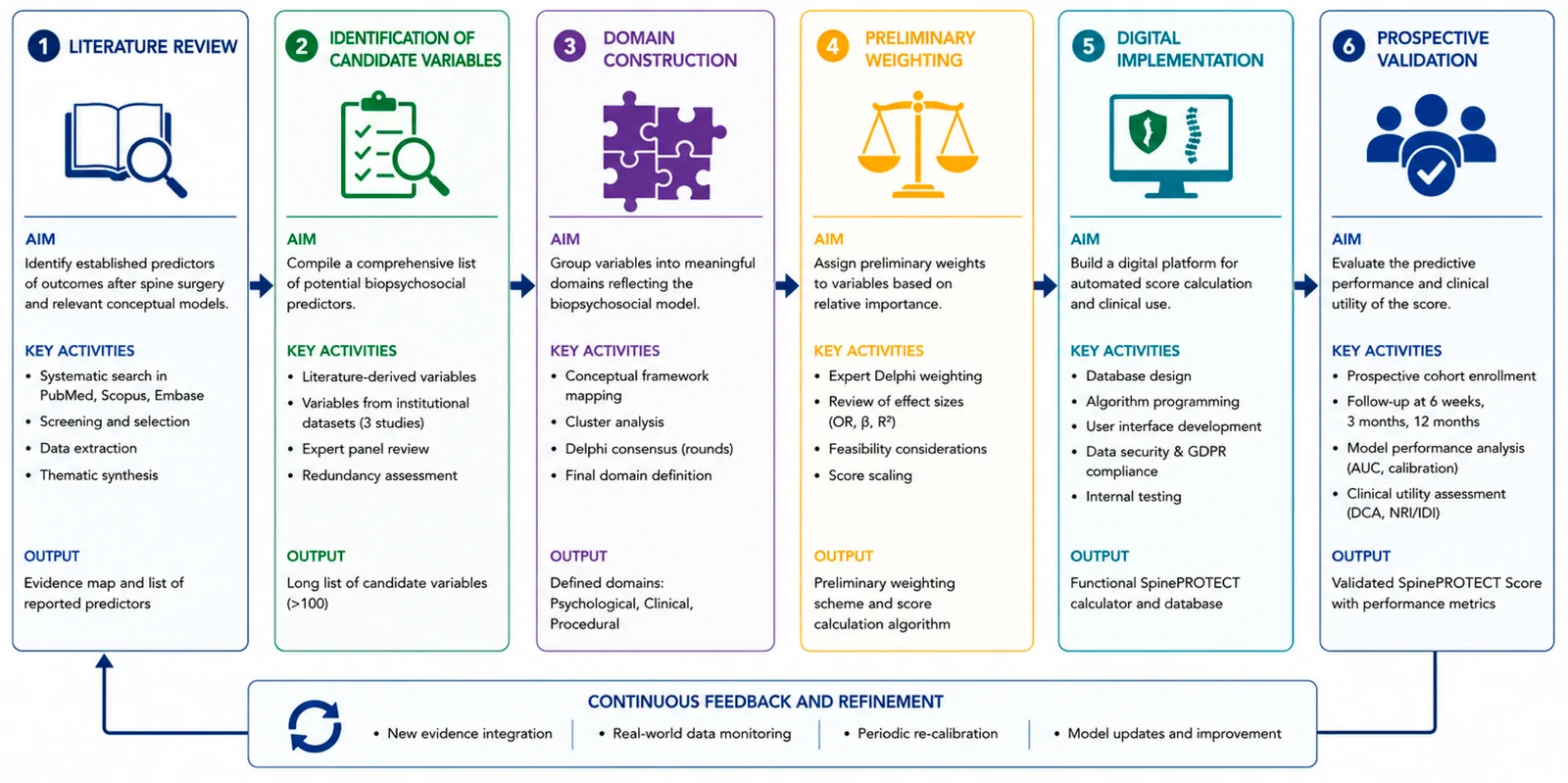
Development process of the SpinePROTECT Score. The framework comprises evidence synthesis, predictor selection, domain construction, preliminary weighting, digital implementation, and prospective model development and internal validation.

The methodological development process is illustrated in Figure 2. Initially, a structured narrative evidence review was performed to identify psychological, clinical, and surgical variables associated with postoperative recovery after spine surgery [1,9,10,11,12,13,14,15,16,31,32,33]. Candidate variables were subsequently compared with findings from our previously completed prospective and cross-sectional clinical investigations [18,19,34].

Preliminary weighting of individual variables was informed by the strength and consistency of available evidence, clinical relevance, reproducibility, and feasibility of routine implementation. These prototype point assignments are hypothesis-generating and will be evaluated descriptively. The prospective cohort will be used to estimate regression coefficients and internally validate the resulting model; it is not an independent external-validation cohort.

After construction of the preliminary scoring framework, the algorithm was translated into a dedicated digital application enabling automated questionnaire acquisition, score calculation, standardized reporting, and longitudinal outcome assessment.

### 2.5. Digital Development

The preliminary algorithm was translated into a modular web-based research application (current prototype version 1.5) to test clinical workflow feasibility. Development objectives included secure authentication, pseudonymized data storage, standardized electronic questionnaire administration, automated calculation, report generation, and export to a structured research database. The software output is not intended to replace clinical judgment, establish surgical indication, or provide validated treatment recommendations before completion of model development and validation.

All figures in this manuscript were created specifically for the SpinePROTECT project by the authors and do not reproduce copyrighted third-party graphical material.

### 2.6. Prospective Model-Development and Internal-Validation Protocol

Prospective model development and internal validation are planned at a tertiary academic spine center. The study was prospectively registered in the German Clinical Trials Register (DRKS00041544) on 8 September 2026 before the planned start of recruitment on 10 September 2026. The study protocol received ethics approval on 28 August 2026 (protocol code 151/26). The observational cohort will include 300 consecutively recruited adults (≥18 years) undergoing elective primary or revision surgery for degenerative disorders of the cervical, thoracic, or lumbar spine. Participants must be able to provide informed consent, complete the German-language patient-reported measures, and attend the planned follow-up. The exclusion criteria are emergency surgery; surgery for acute trauma, infection, or tumor; inability to consent; insufficient German-language proficiency; and absence of planned postoperative follow-up. At the time of prospective registration, recruitment was scheduled to begin on 10 September 2026. The present protocol manuscript does not analyze or report prospective cohort outcomes; actual recruitment dates and participant flow will be reported in the completed model-development study.

To address the clinical heterogeneity of the planned cohort, anatomical region (cervical, thoracic, or lumbar), principal diagnosis, procedure category, surgical approach, number of operated levels, and primary versus revision status will be recorded prospectively as case-mix variables. Revision status is a prespecified candidate predictor within the SpinePROTECT framework. The remaining case-mix variables will be considered for adjustment and descriptive or sensitivity analyses according to the available information and effective sample size. Primary and revision procedures will additionally be described separately and compared in sensitivity analyses; separate subgroup-specific prediction models will not be fitted unless the observed sample size and event count provide adequate statistical information.

Patients complete the full SpinePROTECT assessment preoperatively and undergo standardized follow-up at 6 weeks, 3 months, and 12 months. The primary outcome is failure to achieve a clinically meaningful improvement in ODI at 12 months, defined a priori as an absolute ODI decrease of <12.8 points [37]. Sensitivity analyses will examine a 10-point absolute improvement and a ≥30% relative improvement. Secondary outcomes include ODI and 0–10 numerical rating scale pain scores over time; the four-level Patient Satisfaction Index at 3 and 12 months; opioid use at 3 and 12 months; return to work among participants employed preoperatively; and prospectively recorded treatment-requiring adverse events, reoperations, and revision surgery using prespecified case-report definitions. The planned study endpoints and corresponding assessment timepoints are summarized in Table 2.

The 12.8-point ODI threshold was prespecified before model fitting as a pragmatic anchor for clinically meaningful functional improvement based on the cited lumbar-spine literature [37]. Because published estimates of clinically important ODI change vary across populations, procedures, and baseline disability, the primary definition will not be interpreted as a universal threshold across all spinal regions. The prespecified 10-point absolute and ≥30% relative-improvement sensitivity analyses, together with analysis of ODI as a continuous longitudinal outcome, are intended to evaluate the robustness of findings to alternative definitions of clinically meaningful recovery.

The primary objective is to develop and internally validate a multivariable model for unfavorable 12-month functional recovery. The prototype point score will be evaluated descriptively, whereas regression coefficients will be estimated from the cohort and optimism-corrected through bootstrap resampling. Secondary objectives include evaluation of discrimination, calibration, clinical usefulness, and longitudinal predictive accuracy.

Analyses will include multivariable regression modeling, assessment of discrimination and calibration, bootstrap internal validation, mixed-effects longitudinal modeling, and decision-curve analysis, consistent with contemporary methodological recommendations [29,38].

**Figure 3:**
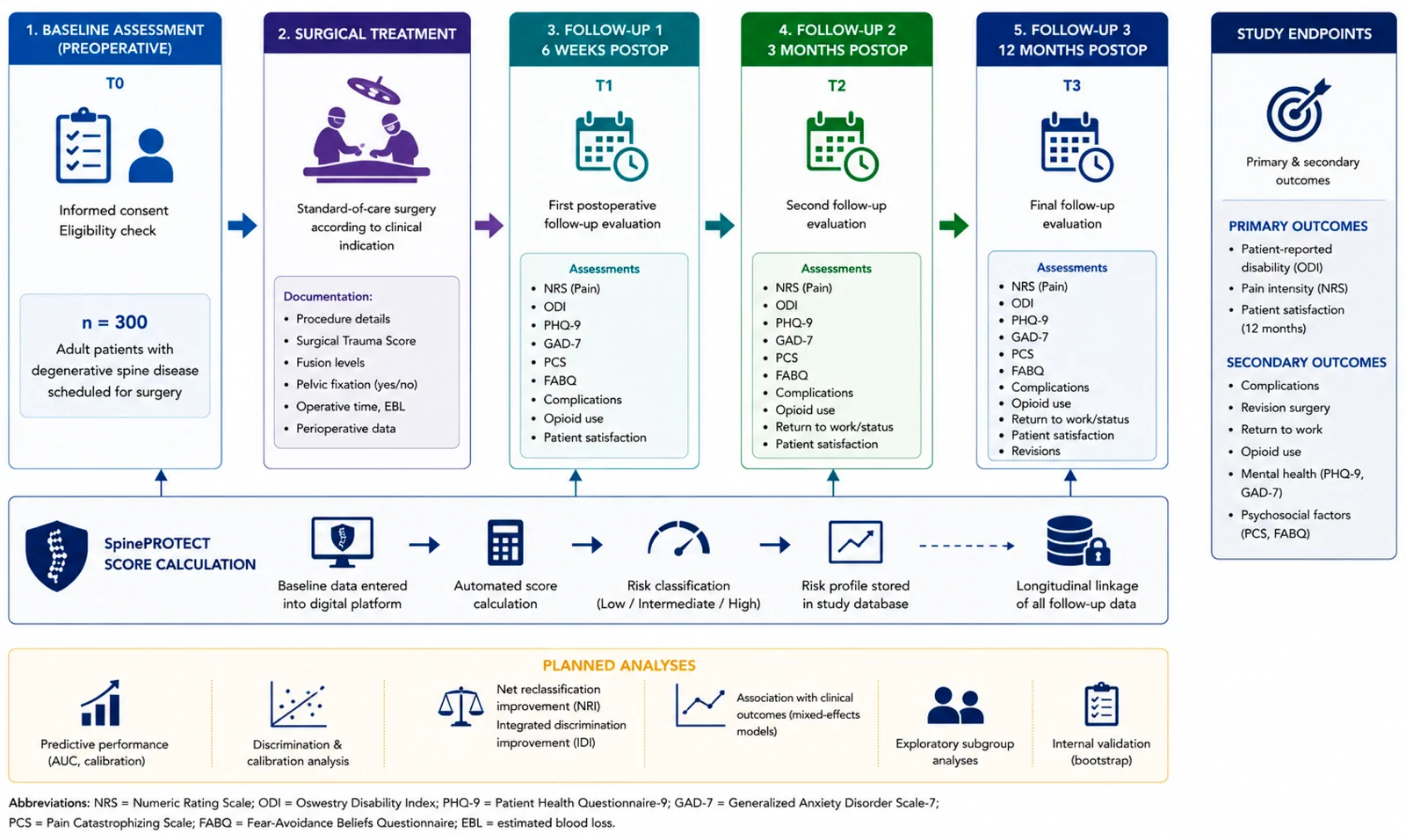
Prospective model-development and internal-validation workflow. Assessments are scheduled preoperatively and at 6 weeks, 3 months, and 12 months after surgery. The colored solid arrows indicate the chronological sequence of the study phases, with colors used to distinguish the individual assessment stages. The dark-blue solid arrows represent the sequential steps of digital score calculation and data processing, whereas the dashed arrow indicates longitudinal linkage of the stored risk profile with follow-up data. The vertical arrows connect the assessment timepoints to the digital data workflow.

The overall model-development and internal-validation workflow is presented in Figure 3.

### 2.7. Statistical Analysis Plan

The present manuscript describes the methodological development of the SpinePROTECT framework and the protocol for prospective model development and internal validation; therefore, no predictive-performance analyses are reported.

The primary binary outcome will be modeled using multivariable regression. Discrimination will be summarized by the area under the receiver operating characteristic curve, calibration by calibration-in-the-large, calibration slope, and graphical calibration, and overall performance by the Brier score. Internal validation and optimism correction will use bootstrap resampling. Mixed-effects models will evaluate longitudinal patient-reported outcomes. Time-to-event outcomes, including revision surgery, will be analyzed using Kaplan–Meier methods and Cox regression where appropriate. Clinical usefulness will be explored with decision-curve analysis.

The prototype point score and the prospective multivariable prediction model will be treated as analytically distinct. The thresholds shown in Table 3 are used only for the exploratory prototype point score and interface/workflow testing and will not automatically be transferred to the multivariable model. During model development, continuous predictors, including PHQ-9, GAD-7, PCS, FABQ, and ODI, will preferentially be retained on their continuous scales; non-linearity will be assessed and modeled flexibly where supported by the available information. The established DS14 definition of Type-D personality (negative affectivity ≥10 and social inhibition ≥10) will remain a prespecified instrument-based classification. Collinearity will be assessed. Missingness will be described by variable and timepoint; if assumptions are tenable, multiple imputation using chained equations will be used. The prototype calculator will assign no risk category when a required core item is missing. The target of 300 participants is pragmatic and is expected to yield approximately 90 primary events if the unfavorable-outcome frequency is about 30%. Before final model fitting, model complexity will be checked against contemporary prediction-model sample-size principles, considering the observed outcome proportion, effective number of candidate parameters, anticipated model fit, shrinkage, and optimism [38,39]. The full cohort will be used for model development; no random training/test split is planned. Internal validation and optimism correction will use bootstrap resampling. If the observed information is insufficient for the prespecified candidate parameter set, model complexity will be reduced or recruitment extended before final model fitting. All changes to predictor handling, weighting, or outcome definitions will be documented transparently.

**Table 3:** Preliminary components and weighting strategy of the SpinePROTECT prototype point score. Point assignments and thresholds are provisional, are used only for exploratory interface/workflow testing, and will not automatically be transferred to the prospective multivariable prediction model. Continuous predictors will preferentially be retained on their original scales during model development.

Domain	Variable	Criteria	Points
Psychological	Type-D Personality	Negative affectivity ≥10 and social inhibition ≥10	+2
Psychological	Depression	PHQ-9 ≥10	+1
Psychological	Anxiety	GAD-7 ≥10	+1
Psychological	Pain Catastrophizing	PCS ≥30	+1
Psychological	Fear Avoidance	FABQ subscale retained as a continuous predictor; no prototype point assignment	Not assigned in the prototype point score
Clinical	Symptom Duration	>12 months	+1
Clinical	Preoperative Opioid Use	Current therapy	+2
Clinical	Baseline Disability	ODI ≥40	+1
Clinical	Revision Surgery	Planned revision	+2
Surgical	Moderate invasiveness	Prespecified STS moderate category [34]	+1
Surgical	High invasiveness	Prespecified STS high category [34]	+2

### 2.8. Ethics, Consent, and Data Protection

The prospective model-development and internal-validation study was approved by the Ethics Committee of the Medical Faculty of Otto von Guericke University Magdeburg on 28 August 2026 (protocol code 151/26). Written informed consent will be obtained before study participation. Data will be collected and stored in pseudonymized form in accordance with institutional data-protection requirements and applicable European and German regulations.

## 3. Current Framework Development Outputs

### 3.1. Preliminary Biopsychosocial Domain Architecture

The structured development process yielded three complementary domains of postoperative recovery: psychological vulnerability, clinical burden, and procedural invasiveness. These domains were combined in a single conceptual framework rather than treated as independent screening instruments.

**Figure 4:**
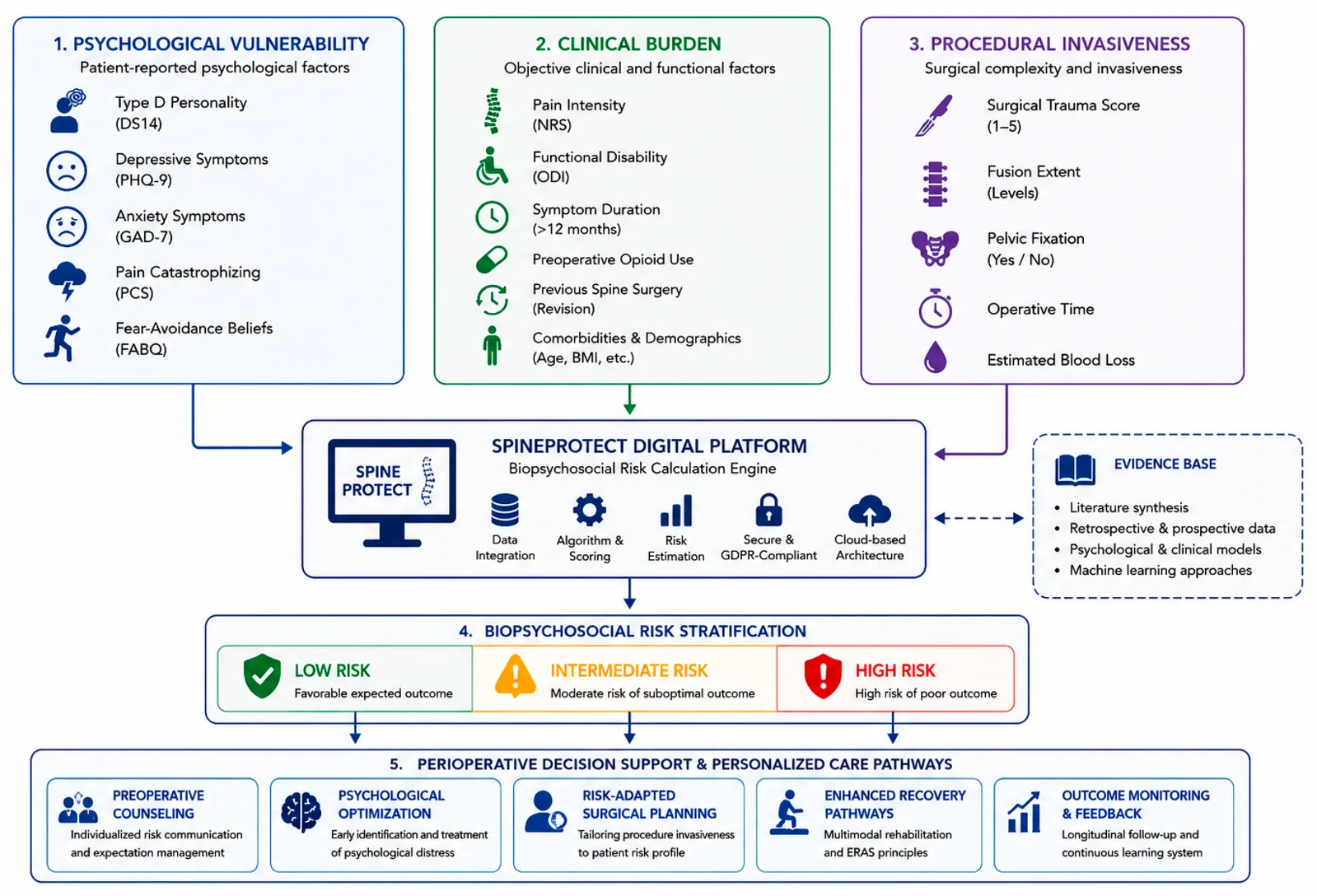
Conceptual overview of the SpinePROTECT framework. Psychological vulnerability, clinical burden, and procedural invasiveness are integrated within a digital perioperative risk stratification pathway. Solid colored arrows represent the contribution of the three predictor domains, the dashed bidirectional arrow indicates the conceptual relationship with the evidence base, and the downward arrows illustrate the subsequent workflow toward preliminary risk stratification and perioperative care pathways.

The development process resulted in a three-domain architecture comprising psychological vulnerability, clinical burden, and procedural invasiveness. The resulting set of prototype components included five psychological measures, four clinical variables, and the Surgical Trauma Score. The resulting architecture is illustrated in Figure 4, and the provisional point assignments are summarized in Table 3.

### 3.2. Preliminary Score Architecture

The preliminary SpinePROTECT framework comprises three complementary domains representing distinct determinants of postoperative recovery: psychological vulnerability, clinical burden, and surgical invasiveness.

Psychological variables contribute substantially to the overall score because they represent potentially modifiable determinants of postoperative recovery and have repeatedly demonstrated prognostic relevance across multiple spine surgery cohorts [10,11,12,13,14,18,19,32,35,40]. Clinical variables account for disease chronicity and baseline patient characteristics known to influence rehabilitation potential [2,16,36]. Surgical variables quantify the biological impact of the planned procedure and complement patient-related risk factors through standardized assessment of operative invasiveness [34].

At the present developmental stage, variable weighting was established using evidence synthesis and expert consensus derived from the available literature and previous institutional investigations. Final weighting coefficients and risk thresholds will be determined during prospective model development and internal validation using multivariable regression modeling, assessment of discrimination and calibration, and bootstrap-based internal validation [29,30,38]. Methodological quality and potential sources of bias were considered throughout model development according to the Prediction model Risk-of-Bias Assessment Tool (PROBAST), particularly regarding participant selection, predictor definition, outcome assessment, and planned statistical validation.

For interface and workflow testing, the prototype platform is capable of displaying three provisional labels: low, intermediate, and high risk. The corresponding thresholds are not validated and are not used for clinical decision-making. Thresholds for the final model will be prespecified or derived using the prospective cohort and subsequently evaluated through internal validation. Preliminary weighting was assigned according to current evidence and expert consensus (Table 3). Final weighting and calibration will be determined during prospective validation using multivariable regression modeling and internal validation procedures.

### 3.3. Digital Platform Output

**Figure 5:**
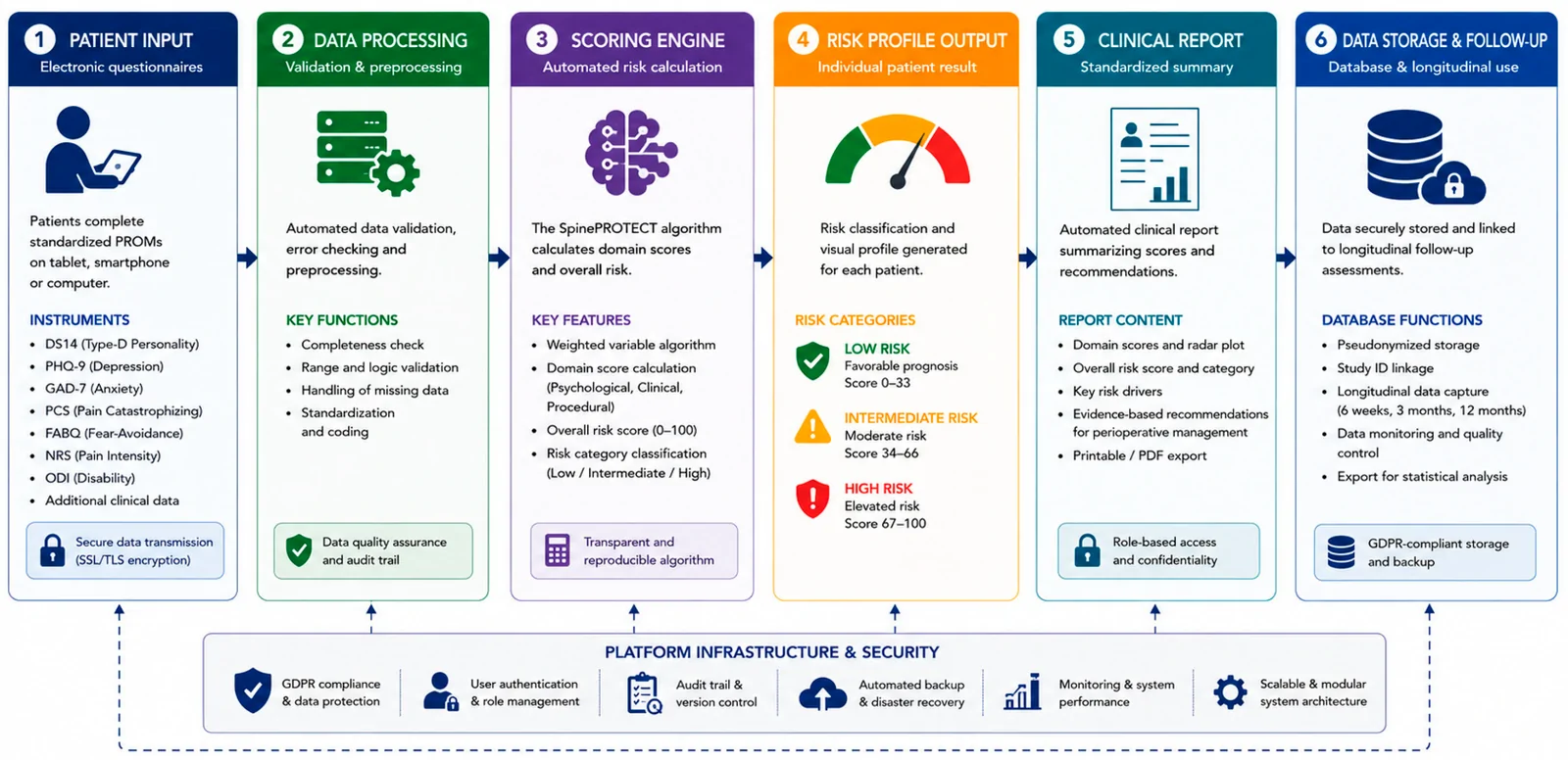
Digital architecture of the SpinePROTECT platform. Electronic questionnaires are processed by a preliminary scoring engine, summarized in a clinical report, and stored for longitudinal research follow-up. Solid horizontal arrows represent the sequential digital workflow, whereas dashed arrows indicate the connection of the individual modules to the shared technical infrastructure and security framework. The different module colors are used for visual differentiation only.

To facilitate research workflow testing, the SpinePROTECT framework was translated into a dedicated digital platform (Figure 5).

The platform was developed as a modular web-based application allowing secure user authentication, encrypted pseudonymized data storage, standardized electronic questionnaire administration, automated score calculation, generation of structured clinical reports, and longitudinal follow-up within a dedicated research database.

Completed questionnaires are processed by an integrated scoring engine that calculates the preliminary SpinePROTECT Score. The prototype can display provisional three-level labels for interface testing, but these labels are not validated, do not trigger treatment recommendations, and will be locked before analysis.

The platform generates standardized research reports summarizing questionnaire results and the provisional risk profile. It does not provide validated treatment recommendations. Data are stored within a structured research database permitting longitudinal outcome monitoring and export for statistical analyses.

The modular software architecture was intentionally designed to support future integration of electronic health records, multicenter databases, wearable devices, and explainable machine-learning algorithms while preserving transparency of the underlying evidence-based framework [21,22,27,28]. Formal usability testing will be performed during prospective clinical implementation to evaluate feasibility, user acceptance, workflow integration, and practical applicability within routine outpatient care. The source code is not publicly released because the platform is a research prototype that processes protected clinical data; technical documentation may be available from the corresponding author subject to institutional and data-security approval.

### 3.4. Current Validation Status

At prospective registration on 8 September 2026, recruitment for model development and internal validation was scheduled to begin on 10 September 2026. The target sample is 300 participants. The present protocol article does not report or analyze prospective cohort outcomes. Definitive regression coefficients, thresholds, discrimination, calibration, and clinical-utility estimates are therefore not available, and no claim of validated predictive performance or external validation is made. Actual recruitment dates and participant flow will be reported in the completed model-development publication.

## 4. Discussion

### 4.1. Principal Findings

The present study describes the scientific rationale, methodological development, and digital implementation of the preliminary SpinePROTECT Score, a biopsychosocial risk stratification framework for spine surgery. Statistical model development and internal validation have been planned according to contemporary reporting and risk-of-bias guidance [29,30] and prediction-model methodology [38]. The platform integrates validated psychological patient-reported measures, clinical risk factors, and standardized assessment of surgical invasiveness within one digital research workflow. Because the present work is a protocol and framework development report, any claim of comparative superiority would require direct comparative evaluation and is not made here.

The SpinePROTECT framework is based on the premise that postoperative recovery reflects interactions between biomedical and psychosocial factors [1,3,8]. Psychological distress, catastrophizing, fear-related beliefs, and expectations have each been linked to pain, disability, satisfaction, or other recovery outcomes in spine or musculoskeletal populations [9,10,11,12,13,14,15,31,32,33]. The framework development process additionally incorporated our institutional research on Type-D personality and procedural invasiveness [18,19,34]. Its prospective model-development strategy follows contemporary recommendations for transparent reporting, risk-of-bias assessment, and internal validation [29,30,38].

Importantly, the objective of the SpinePROTECT framework is not to replace established surgical indications or surgeon expertise. If validated, individualized risk estimates could instead complement clinical decision-making and support counseling or risk-adapted perioperative planning. Prediction tools have been proposed as aids to prognostication and shared decision-making in spine surgery, but their usefulness depends on appropriate validation and implementation [21,41].

If prospectively validated, the intended clinical significance of SpinePROTECT is not to determine whether surgery should or should not be performed. Rather, individualized risk information could support preoperative counseling, identification of potentially modifiable psychosocial or opioid-related factors, allocation of multidisciplinary perioperative support, and selection of patients who may benefit from intensified postoperative follow-up.

Primary and revision spine surgery represent clinically distinct contexts with different baseline risks, technical complexity, and recovery trajectories. The protocol therefore treats planned revision surgery as a prespecified candidate predictor and will report primary and revision procedures separately in descriptive and sensitivity analyses. This approach allows revision status to contribute prognostic information without assuming that both groups have identical baseline risk structures.

### 4.2. Comparison with Existing Prediction Models

Prediction models are increasingly reported in spine surgery and address outcomes ranging from complications and frailty-associated risk to patient-reported recovery [20,21,22,24,41]. Reviews show substantial variation in populations, predictors, outcomes, and validation status [20,21,22,41]. Therefore, Table 4 provides a descriptive conceptual comparison rather than a formal systematic comparative assessment.

Psychological determinants are not consistently represented in existing spine prediction models [20,21]. At the same time, psychological distress has demonstrated prognostic relevance in spine surgery cohorts and reviews [1,9,10,11,12,15]. Established instruments such as the DS14, PCS, and FABQ quantify specific psychosocial constructs [17,25,26], but measurement of an individual construct is distinct from development and validation of an integrated multivariable prediction model [29,30].

The SpinePROTECT framework was developed to examine whether combining psychological vulnerability, clinical burden, and procedural invasiveness can improve structured risk stratification. Its psychological component is informed by our Type-D studies [18,19], while operative invasiveness is represented by the Surgical Trauma Score [34]. Preoperative opioid exposure is included because contemporary evidence associates it with prolonged postoperative opioid use and worse spine surgery outcomes [16]. The multidimensional architecture is conceptually consistent with biopsychosocial models in which recovery reflects interacting patient- and treatment-related factors [3,8].

A detailed comparison between the SpinePROTECT framework and representative perioperative prediction models is presented in Table 4.

### 4.3. Methodological and Clinical Implications

The present study also addresses methodological aspects of prediction-model research in spine surgery. The framework was developed through a prespecified sequence of evidence review, candidate-predictor selection, prototype construction, digital implementation, and prospective model-development planning. Transparent reporting and risk-of-bias considerations are informed by TRIPOD+AI and PROBAST [29,30], while sample-size and internal-validation planning follows contemporary prediction-model methodology [38,39].

The framework combines a structured narrative evidence review with institutional observational research on Type-D personality, psychosocial burden, and surgical invasiveness [18,19,34]. Candidate predictors were additionally screened for clinical feasibility, reproducibility, and suitability for standardized prospective data collection. The subsequent statistical model will be developed and internally validated using prespecified methods intended to limit overfitting and quantify optimism [38,39].

A further methodological feature is the prespecified integration of psychosocial, clinical, and procedural candidate predictors. Existing spine prediction models vary widely in their predictor domains, and psychosocial variables are not consistently incorporated [20,21,22,41]. The inclusion of psychological constructs is supported by evidence linking psychological distress, catastrophizing, fear-related beliefs, and expectations with pain, disability, satisfaction, or other postoperative outcomes [9,10,11,12,13,14,15,31,32,33].

The framework was designed from the outset for digital research implementation. In the current prototype, electronic questionnaire administration, automated score calculation, structured reporting, and longitudinal follow-up are implemented as platform functions. Broader integration with routine clinical workflows or electronic health record systems remains a future objective rather than an established capability; the need for external validation and implementation testing is consistent with current reviews of digital and machine-learning prediction models in spine surgery [21,22,27,28].

Finally, model development and internal validation were incorporated as predefined protocol components. Planned reporting, risk-of-bias assessment, and evaluation of model performance follow TRIPOD+AI, PROBAST, and contemporary prediction-model methodology [29,30,38]. External validation will remain necessary because internal validation alone cannot establish transportability to other settings; recent multicenter work in lumbar fusion illustrates the importance of evaluating both discrimination and calibration in independent data [42].

Preoperative risk stratification is relevant because postoperative outcomes are associated with multiple patient-level domains. Frailty is associated with adverse events, mortality, length of stay, readmission, and non-home discharge after spine surgery [24]. Psychological distress is associated with several postoperative outcomes [12,15], and preoperative opioid use is associated with chronic postoperative opioid use and other adverse outcomes [16]. Prediction tools seek to combine such information to support individualized prognostication, although model validity and transportability must be established before routine use [20,21,41,42].

If prospectively validated, SpinePROTECT could have several clinical applications. Quantified prognostic information may support preoperative counseling and shared decision-making, an intended use also described for prediction tools in spine surgery [41]. Identification of psychological vulnerability could help identify patients for whom targeted psychological support merits consideration; a recent systematic review found that several preoperative psychological interventions were associated with improved postoperative outcomes, although the evidence remains heterogeneous [43]. Identification of preoperative opioid exposure may similarly prompt structured counseling because preoperative opioid use is associated with prolonged postoperative use and worse outcomes [16]. Any use for resource allocation or intensified follow-up would require prospective demonstration of clinical utility rather than being assumed from risk classification alone [29].

Importantly, the SpinePROTECT framework is not intended to deny or delay surgical treatment. Its intended role is complementary risk stratification. Psychological and opioid-related factors may be potentially modifiable in selected patients [16,43], but whether modification of these factors improves outcomes within the SpinePROTECT pathway must be tested prospectively.

### 4.4. Digital Translation

A distinctive characteristic of the SpinePROTECT framework is its early translation into a dedicated digital research platform. Reviews of spine prediction models and machine-learning applications indicate that clinical implementation and external validation remain variable and frequently limited [21,22,41]. SpinePROTECT was therefore designed initially to support standardized data collection and prospective research; it must not be used as a validated clinical decision-support system before model development, internal validation, and subsequent external validation are complete.

The current prototype enables automated collection of questionnaires, standardized calculation of the provisional score, structured research-report generation, longitudinal outcome monitoring, and storage within a dedicated research database. These are functions of the developed prototype and are not presented as validated clinical benefits. Formal usability testing during prospective implementation will be required to determine administrative burden, workflow feasibility, and user acceptance.

The modular architecture was designed to permit future interoperability with electronic health records, multicenter registries, and additional digital data sources. Machine-learning and digital prediction approaches are increasingly investigated in spine surgery [22,27,28], but successful integration requires technical validation, data-governance safeguards, and evaluation across independent clinical settings [21,22].

Future machine-learning extensions could be evaluated for model updating or dynamic prediction, but such applications would require transparent reporting, calibration assessment, external validation, and clinically interpretable outputs [22,27,28,29]. These possibilities are therefore presented as future research directions rather than established capabilities of the current SpinePROTECT prototype.

### 4.5. Strengths and Limitations

Several methodological strengths should be acknowledged. First, the SpinePROTECT framework was developed through a transparent process integrating a structured narrative evidence review with three complementary clinical research programs rather than relying solely on expert opinion. Second, it combines validated psychological instruments, clinically established risk factors, and standardized assessment of procedural invasiveness within a unified biopsychosocial model. Third, digital implementation was incorporated early, facilitating standardized prospective data collection and future multicenter research.

This study has important limitations. The proposed point assignments are preliminary, expert-informed, and not derived from regression coefficients; their performance and any risk thresholds remain unknown. The target sample of 300 is pragmatic, and the effective number of candidate parameters will be checked against contemporary prediction-model sample-size principles before final model fitting; model complexity will be reduced or recruitment extended if the observed information is insufficient [38,39]. The planned cohort intentionally includes heterogeneous degenerative cervical, thoracic, and lumbar procedures and both primary and revision surgery. Although this improves clinical breadth, it may introduce case-mix heterogeneity; anatomical region, diagnosis, procedure category, approach, operated levels, and primary/revision status will therefore be documented prospectively, with prespecified descriptive, adjustment, and sensitivity analyses as appropriate. The 12.8-point ODI primary threshold was prespecified and is not assumed to represent a universal MCID across all spinal populations; alternative absolute and relative thresholds and continuous ODI trajectories will be examined in sensitivity and secondary analyses. The structured narrative evidence review was not a formal systematic review. Development is single-center, so internal validation cannot establish transportability; independent multicenter external validation will be required. The prototype categories and reports must not be used to deny, delay, or recommend treatment. Psychosocial assessment remains one component of perioperative evaluation and must be interpreted alongside clinical, radiological, and surgical findings.

### 4.6. Future Perspectives

The present framework provides a foundation for future digital development. After model development and internal validation, dynamic prediction could be investigated as a separate extension in which risk estimates are updated with longitudinal information; machine-learning approaches in spine surgery provide methodological precedent for such future work [22,27,28].

One future direction is integration with electronic health record systems. Automated extraction of routinely collected variables could reduce duplicate data entry, but the magnitude of any workflow benefit must be evaluated empirically. Reviews of digital and machine-learning applications in spine surgery emphasize that translation from model development to validated clinical implementation remains a distinct step [21,22,27,28].

Multicenter implementation could also support external validation and assessment of transportability. Independent validation is essential because model performance may change across populations and healthcare settings [41,42]. A prospective multicenter registry could subsequently provide data for recalibration or model updating if clinically and statistically justified.

Future versions may investigate dynamic prediction using postoperative information such as early recovery trajectories, complications, medication use, and repeated PROM assessments. This would constitute a new model-development step requiring its own validation rather than an automatic extension of the baseline model [29,30].

Wearable sensors, smartphone-based monitoring, and remote PROM collection could also be evaluated as additional data sources in future studies. Their incremental predictive value would need to be demonstrated rather than assumed, with attention to data quality, missingness, and transportability [29,30].

Federated or other privacy-preserving analytic approaches may eventually be relevant to multicenter model updating. Because these methods have not been implemented in the current project, they are considered exploratory future options and would require dedicated technical, methodological, ethical, and data-governance evaluation.

If machine-learning methods are evaluated in future versions, transparency and interpretability should remain explicit design requirements. The current literature describes increasing use of artificial intelligence and machine learning in spine surgery prediction research [27,28], while TRIPOD+AI provides updated reporting guidance for prediction models using regression or machine-learning methods [29].

Taken together, these possibilities define a future research agenda rather than validated functionality. Any transition from the present preoperative research framework to a dynamic clinical decision-support system would require prospective model development, rigorous internal and external validation, demonstration of clinical utility, and evaluation of implementation in independent settings [29,30,38,42].

## 5. Conclusions

The SpinePROTECT project establishes a transparent methodological and digital framework for biopsychosocial risk stratification in spine surgery. It integrates validated psychological measures, clinical characteristics, and procedural invasiveness within a structured platform for prospective evaluation. The preliminary weights and risk categories must not be interpreted as validated predictors. Completion of the prespecified prospective study is required to determine model coefficients, discrimination, calibration, internal validity, and clinical utility before implementation as a clinical decision-support instrument.

## Figures and Tables

**Table 1:** Variable-selection framework and evidence sources underlying the SpinePROTECT Score.

Domain	Variable	Instrument/Assessment	Rationale	Supporting Evidence
Psychological	Type-D Personality	DS14	Stable psychosocial vulnerability profile associated with poorer recovery and greater disability	Denollet [17]; Riediger et al. [18,19]
Psychological	Depression	PHQ-9	Associated with poorer postoperative outcomes	Aghajanian [12]; Harris [15]
Psychological	Anxiety	GAD-7	Associated with increased pain perception and reduced satisfaction	Yamamoto [31]; Harris [15]
Psychological	Pain Catastrophizing	PCS	Amplifies pain processing	Sullivan [25]; Meints [32]
Psychological	Fear Avoidance	FABQ	Predicts disability	Waddell [26]; Leeuw [33]
Clinical	Baseline Disability	ODI	Predictor of postoperative function	Fairbank [36]
Clinical	Symptom Duration >12 months	Clinical history	Associated with poorer recovery	Campbell [35]
Clinical	Preoperative Opioid Use	Medication history	Associated with prolonged opioid dependence	Wang [16]
Clinical	Revision Surgery	Surgical history	Higher complexity	Mannion [2]
Surgical	Surgical Trauma Score	Surgical Trauma Score (STS) derived from the published Surgical Trauma Gradient	Standardizes operative burden	Riediger et al. [34]

**Table 2:** Planned prospective model-development study and outcome measures.

Endpoint Category	Outcome Measure	Assessment Timepoint
Primary	Failure to achieve an absolute ODI decrease of ≥12.8 points, operationalized as an ODI decrease of <12.8 points at 12 months	12 months
Secondary	ODI	Baseline, 6 weeks, 3 months, 12 months
Secondary	Pain intensity (0–10 NRS)	Baseline, 6 weeks, 3 months, 12 months
Secondary	Four-level Patient Satisfaction Index	3 and 12 months
Secondary	Ongoing opioid use	3 and 12 months
Secondary	Return to work (participants employed preoperatively)	3 and 12 months
Secondary	Treatment-requiring adverse events	Continuous
Secondary	Reoperation or revision surgery	Continuous

**Table 4:** Descriptive comparison of representative perioperative prediction approaches and the SpinePROTECT framework. The table summarizes typical areas of emphasis reported in representative descriptions and reviews [20,21,22,24,41] and is intended for contextual orientation rather than ranking, scoring, or formal systematic comparison.

Approach	Typical Primary Focus	Typical Predictor Domains	Psychosocial Content	Procedural/Clinical Content	Implementation/Validation Context
SpineSage-type models	Perioperative complications	Demographic, comorbidity, and procedure-related variables	Not a defining feature of the representative approach	Procedure complexity and clinical comorbidity are central	Published risk-calculator approaches; validation depends on model/version
ACS NSQIP-based approaches	General postoperative complications	Demographic, comorbidity, laboratory, and procedural variables	Not a defining feature of the general surgical calculator	Broad perioperative clinical and procedural variables	Web-based/general surgical risk-calculator framework with published validation
Frailty approaches	Frailty-associated perioperative risk	Frailty, functional reserve, comorbidity, and demographic variables	May be represented indirectly depending on frailty instrument	Patient reserve and clinical burden are central	Multiple instruments and cohorts; validation is model-specific
PROM prediction models	Patient-reported postoperative outcomes	Model-specific combinations of demographic, clinical, procedural, and PROM variables	Variable; depends on the individual model	Clinical/procedural content varies by outcome and model	Heterogeneous models; external validation and implementation vary
SpinePROTECT framework	Biopsychosocial risk stratification for unfavorable recovery	Prespecified psychological, clinical, and procedural candidate predictors	DS14, PHQ-9, GAD-7, PCS, and FABQ are explicitly incorporated	Symptom duration, ODI, opioid exposure, revision status, and Surgical Trauma Score	Dedicated research platform; prospective model development/internal validation planned; external validation required

## Data Availability

No validation dataset is reported in this protocol article. Data from the underlying institutional studies are not publicly available because of participant confidentiality and institutional data-protection requirements; de-identified data may be available from the corresponding author upon reasonable request and subject to ethics and institutional approval. Data from the prospective cohort will be addressed in the subsequent model-development report. Source code is not publicly available because the research prototype processes protected clinical data; technical documentation may be requested from the corresponding author subject to institutional approval.

## Abbreviations

The following abbreviations are used in this manuscript:

**Abbreviation**	**Definition**
ACS NSQIP	American College of Surgeons National Surgical Quality Improvement Program
AI	Artificial Intelligence
AUC	Area Under the Receiver Operating Characteristic Curve
CI	Confidence Interval
DS14	Type-D Scale-14
FABQ	Fear-Avoidance Beliefs Questionnaire
GAD-7	Generalized Anxiety Disorder-7
ML	Machine Learning
ODI	Oswestry Disability Index
PCS	Pain Catastrophizing Scale
PHQ-9	Patient Health Questionnaire-9
PROM	Patient-Reported Outcome Measure
PRO	Patient-Reported Outcome
ROC	Receiver Operating Characteristic
STS	Surgical Trauma Score
VAS	Visual Analog Scale
